# Whole-cycle management of women with epilepsy of child-bearing age: ontology construction and application

**DOI:** 10.1186/s12911-024-02509-z

**Published:** 2024-04-18

**Authors:** Yilin Xia, Yifei Duan, Leihao Sha, Wanlin Lai, Zhimeng Zhang, Jiaxin Hou, Lei Chen

**Affiliations:** 1 Department of Neurology, West China Hospital, Sichuan University, #37 Guoxue Alley, Wuhou District, 610041 Chengdu, Sichuan Province China; 2 Pazhou Lab, Guangzhou, China

**Keywords:** Epilepsy, Ontology, Women health, Online question-and-answer system

## Abstract

**Background:**

The effective management of epilepsy in women of child-bearing age necessitates a concerted effort from multidisciplinary teams. Nevertheless, there exists an inadequacy in the seamless exchange of knowledge among healthcare providers within this context. Consequently, it is imperative to enhance the availability of informatics resources and the development of decision support tools to address this issue comprehensively.

**Materials and methods:**

The development of the Women with Epilepsy of Child-Bearing Age Ontology (WWECA) adhered to established ontology construction principles. The ontology’s scope and universal terminology were initially established by the development team and subsequently subjected to external evaluation through a rapid Delphi consensus exercise involving domain experts. Additional entities and attribute annotation data were sourced from authoritative guideline documents and specialized terminology databases within the respective field. Furthermore, the ontology has played a pivotal role in steering the creation of an online question-and-answer system, which is actively employed and assessed by a diverse group of multidisciplinary healthcare providers.

**Results:**

WWECA successfully integrated a total of 609 entities encompassing various facets related to the diagnosis and medication for women of child-bearing age afflicted with epilepsy. The ontology exhibited a maximum depth of 8 within its hierarchical structure. Each of these entities featured three fundamental attributes, namely Chinese labels, definitions, and synonyms. The evaluation of WWECA involved 35 experts from 10 different hospitals across China, resulting in a favorable consensus among the experts. Furthermore, the ontology-driven online question and answer system underwent evaluation by a panel of 10 experts, including neurologists, obstetricians, and gynecologists. This evaluation yielded an average rating of 4.2, signifying a positive reception and endorsement of the system’s utility and effectiveness.

**Conclusions:**

Our ontology and the associated online question and answer system hold the potential to serve as a scalable assistant for healthcare providers engaged in the management of women with epilepsy (WWE). In the future, this developmental framework has the potential for broader application in the context of long-term management of more intricate chronic health conditions.

**Supplementary Information:**

The online version contains supplementary material available at 10.1186/s12911-024-02509-z.

## Introduction

Epilepsy is a chronic disease of the nervous system with heterogeneous characteristics. Patients presenting with varying epilepsy characteristics necessitate personalized approaches to disease management. One notable example of the complexity in managing epilepsy is observed in women with epilepsy (WWE) during their child-bearing years. Upon entering puberty, roughly 40% of WWE experience catamenial epilepsy, a condition marked by heightened seizure activity correlated with hormonal fluctuations throughout the menstrual cycle [1]. It is worth noting that there are approximately 25 million WWE worldwide, and approximately one-third of this population falls within the child-bearing age bracket [2].

However, the use of some anti-seizure medications (ASMs)[but not all] can cause reproductive-endocrine dysfunction, leading to complications like polycystic ovary syndrome and infertility [3]. Furthermore, WWE are at risk of unplanned pregnancy, including potentially due to low awareness regarding the need of planning pregnancy, as well as the adverse interactions between ASMs and contraceptives [4]. Moreover, WWE often experience fluctuating ASMs levels during pregnancy, primarily due to physiological increases in blood volume, which can lead to inadequate epilepsy management. Additionally, WWE on anti-epileptic medications face an elevated risk of major fetal malformations [5]. Beyond these considerations, WWE may face a heightened risk of adverse pregnancy outcomes, including preeclampsia, preterm birth, stillbirth, and maternal mortality [5–7]. Some of ASMs in breastfeeding WWE may result in neurodevelopmental disorders without an identifiable malformation [3, 8, 9]. In addition to these physical challenges, women with epilepsy of child-bearing age contend with additional societal and familial pressures, heightened stigma, and a lower quality of life compared to their male counterparts [10].

Given the intricacies involved in the treatment of WWE in their child-bearing years, a multidisciplinary team approach (MDT) becomes essential. This approach typically comprises a range of specialists, including epileptologists, gynecologists, reproductive endocrinologists, obstetricians, pharmacists, and ultrasonographers. However, it’s important to note that in many countries and regions with less advanced medical infrastructure, this comprehensive and resource-intensive management model may not be readily available, making it challenging to achieve standardized care. To address this disparity, over ten epilepsy guidelines worldwide offer more than a hundred and fifty management recommendations, which could serve as an initial step in bridging the gap between guideline advice and practical clinical implementation.

Nonetheless, there exists a significant disparity between the guidance outlined in these written recommendations and the efficient, consistent application of these principles in clinical practice. To address this issue, healthcare providers require improved resources and tools. In this context, an ontology emerges as a potent instrument to enhance multidisciplinary interoperability, facilitating rapid access to knowledge for healthcare professionals and providing patients and their families with access to high-quality information. The concept of ontology originally finds its roots in Western philosophy, denoting the systematic description of an objective entity. Over recent decades, it has been adopted in the realm of informatics, and its definition has evolved to encompass “a formal specification of terms and their interrelationships” [11]. In the biomedical domain, ontologies play a transformative role in the integration, interaction, and dissemination of complex information.

Therefore, we are embarking on the creation of an ontology tailored to women of child-bearing age with epilepsy (WWECA), drawing from guideline documents and expert opinions concerning WWE during their child-bearing years. Additionally, we are developing an ontology-driven question-and-answer system, with the aspiration of enhancing consistency and standardization in the management of WWE in their child-bearing years worldwide, particularly in low- and middle-income countries.

## Methods

**Fig. 1:**
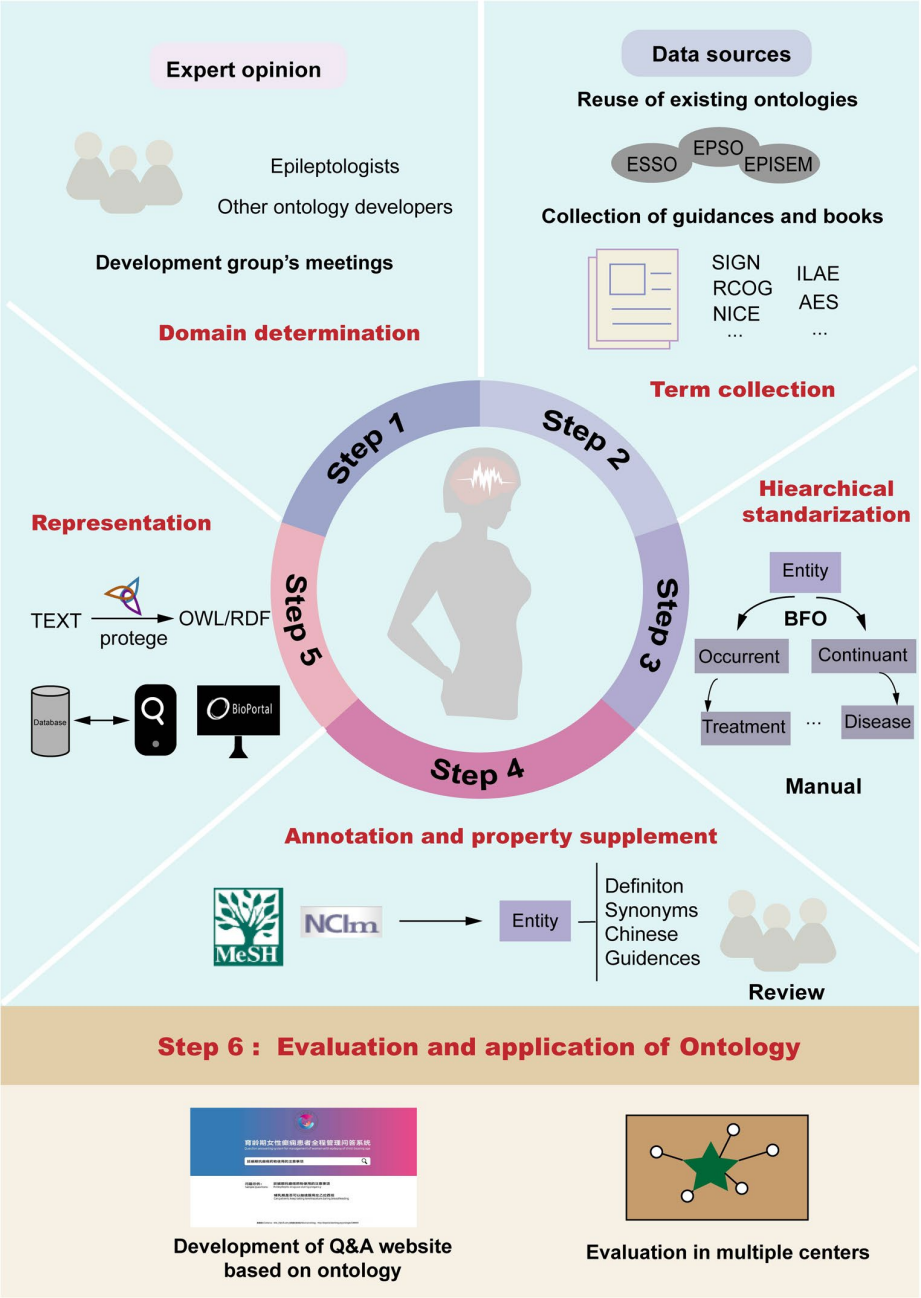
Workflow of construction for women with epilepsy of child-bearing age. Step1 development team scoped the ontologynd identifies universal terminology; Step2 Data was collected from existing ontologies and authoritative guidelines in the field; Step3 The terminology hierarchy was arranged, and relationships were defined. Step 4 The ontology was annotated and compiled; Step5 Protégé (open source ontology editor and knowledge-based framework) formally constructed the ontology; Step6 Ontology-driven applications were developed and evaluated; (Abbreviations: ESSO: Epilepsy Syndrome Seizure Ontology, EPSO: Epilepsy and Seizure Ontology, EPISM: Epilepsy Semiology)

We followed the general principles and methods of ontology construction to design this domain ontology for the treatment of WWE of child-bearing age [12]. As shown in Fig. 1, construction consists of six main stages.

### Scope and data collection

The scope of the ontology, the upper-level entities, and the attributes of the entities were defined by the development team through offline face-to-face meetings [13]. Definitions and synonyms were collected from the authoritative glossary Medical Subject Headings (MeSH, version date: 1 December 2021) and National Cancer Institute metathesaurus (NCIm, version number: 2.19).The literature was searched from 2000 to 2021 utilizing a combination of the following keywords: “epilepsy,” “guidelines,” “consensus,” and “expert opinion” in databases including PubMed, International Guidelines Library (GIN, https://g-i-n.net/), National Institute for Health and Care Excellence (NICE, https://www.nice.org.uk/), Scottish Intercollegiate Guidelines Network (SIGN, https://www.sign.ac.uk/), Royal College of Obstetricians and Gynecologists Guidelines Library (RCOG, https://www.rcog.org.uk/), and the Chinese Medical Guidelines Library. The primary criterion for selection was the relevance of guidelines pertaining to women with epilepsy. Subsequently, a systematic knowledge elicitation approach was employed to extract terms representing ASMs and multiple reproductive age stages from the guidelines, added it to the ontology as an additional annotation of the corresponding entity, and named the annotation with the abbreviation of the source guideline name. Furthermore, in expanding the ontology, we utilized existing epilepsy ontologies, reference domain guidelines, and relevant literature sources to enrich the entity descriptions and relationships within the ontology.

### Hierarchical construction and ontological representation

To ensure the interoperability of different domain knowledge, we chose the Basic Formal Ontology (BFO) as the top-level ontology. BFO classifies entities in ontologies into the categories of “constants” and “actors”: constants are entities that persist through time, while actors are entities that occur or appear, also known as “events” or “processes” or “things that happen“ [14]. The entities are arranged in the “is_a” relation down the hierarchy, resulting in a directed acyclic graph with a single root node. The development team worked together in biweekly meetings throughout the development process.We used Protégé (version 5.5.0) to formalize concepts and relationships in OWL (Web Ontology Language) as the format for outputting the ontology).

### Application development and evaluation of ontology

We uploaded the ontology to the NCBO Bioportal to make the ontology generally available. We also developed a stand-alone web-based question and answer system using HTML5 and ASP.net, using various methods such as reverse maximum match (RMM), bidirectional matching method (BM), N-gram algorithm, Hidden Markov Model (HMM) algorithm, etc., which supports users to be able to ask bilingual (Chinese and English) questions about the use of antiseizure medication (ASMs) in women with epilepsy of child-bearing age. Relevant details of the website query algorithm can be found in the Supplementary Materials(sTable 3 and video).

The external assessment of WWECA involved four distinct methods. Firstly, We invited informaticians and experts from the multidisciplinary female epilepsy team (experienced obstetricians and gynaecologists, reproductive endocrinologists, paediatricians) to evluate the accuracy, completeness, clarity, consistency and conciseness of the ontology [15].An introductory session took place at the beginning of the interview. The concept of ontology was explained to the experts through examples from the biomedical field.

We extended invitations to epileptologists nationwide in China to engage in a rapid Delphi consensus exercise. In the first round, we shared the initial version of our ontology with epileptologists and informaticists, requesting their input on any concepts that were absent from the ontology but were integral to their clinical workflows [16]. In the second round, we disseminated a revised version of the ontology to a larger group of epileptologists and employed a 5-point Likert scale (ranging from 1 to 5) to gauge their level of agreement regarding the ontology’s completeness. A consensus was defined as having achieved a level of agreement of ≥ 80%. Those aspects that fell short of this threshold were subject to revision based on the feedback received. For the third round, we organized an online discussion involving all assessment experts to collectively review and endorse the final version of the ontology. Online link for participation in the survey was sent to all of the experts by a well-known Chinese survey website WenJuanXing.

Additionally, we invited attending physicians, residents, and interns from the fields of neurology, obstetrics, and gynecology to utilize our self-developed web-based question and answer system. Subsequently, we conducted a satisfaction survey using a 5-point Likert scale, where higher scores signified a greater degree of consensus on the system’s effectiveness. It is important to note that the study underwent a rigorous review and received approval from the Ethics Committee of West China Hospital at Sichuan University. Written informed consent was obtained from all participants involved in the assessment. Finally, we evaluated and validated WWECA on six levels based on Brank’s research [17].

## Results

### Scope of the ontology and data sources

The scope of ontology was established through consensus during a series of seven meetings within the development team, and dynamically adapted and refined after evaluation. It was defined as encompassing the management of epilepsy and the maintenance of reproductive health among women with epilepsy (WWE) throughout their reproductive years. This inclusive scope covered various life stages, including adolescence, pregnancy, childbirth, and lactation. The primary intended users of the ontology are healthcare providers, which includes specialists such as neurologists, obstetricians, and gynecologists, etc. Additionally, the ontology is designed to benefit neuroinformatic researchers specializing in the field of WWE of child-bearing age(sTable 2).

**Fig. 2:**
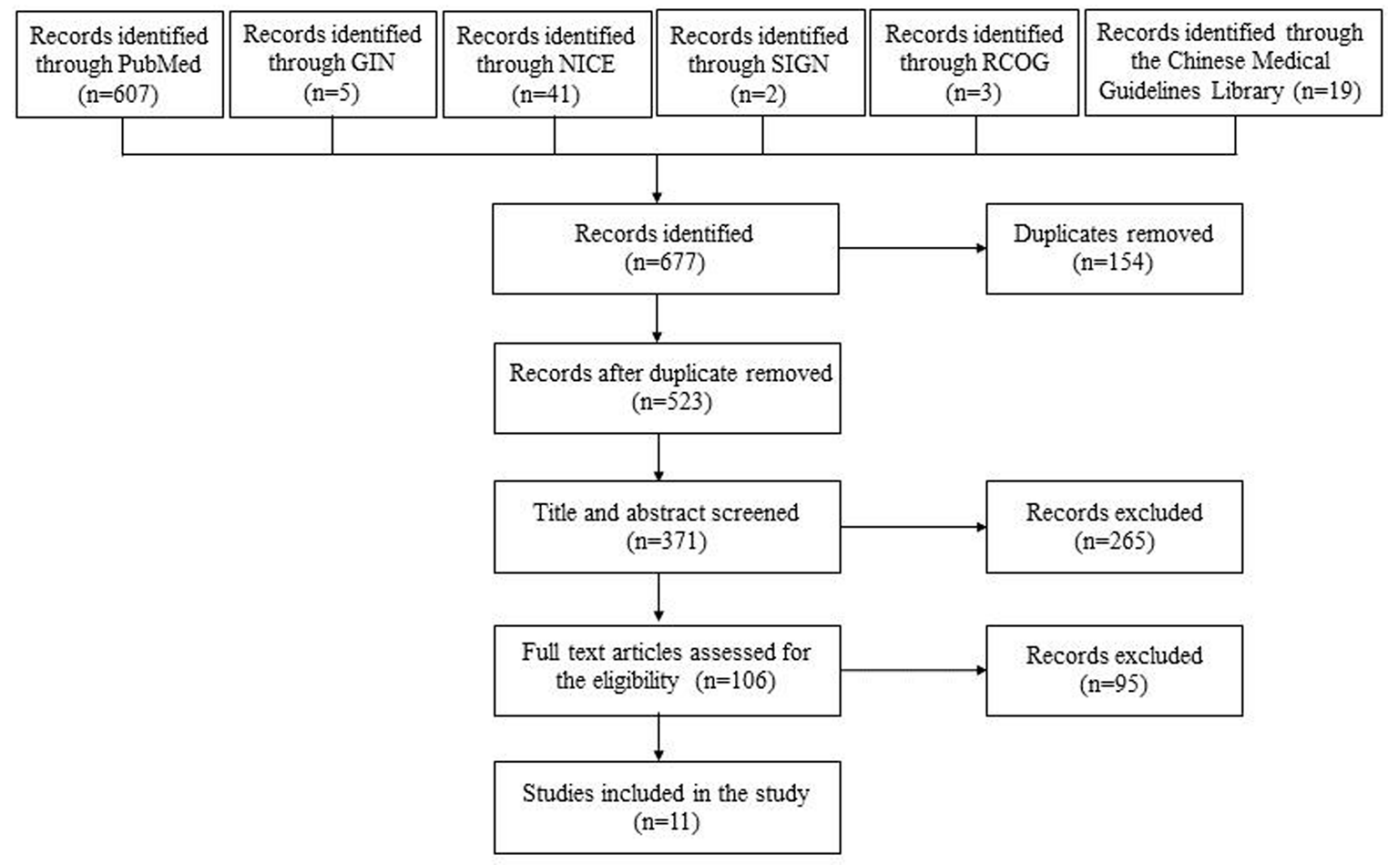
Flowchart of the publication search process

The prevalent terms extracted from the key questions posed by the epilepsy experts on the development team accounted for 32.4% of the final entities, mainly the superordinate entities of the ontology. Part of the other entities were reused from an existing ontology *Epilepsy and Seizure Ontology (EPSO)* [18]. The guidelines we refer to include *Epilepsies: diagnosis and management* from the National Institute for Health and Care Excellence, *Epilepsy in Pregnancy* from the Royal College of Obstetricians & Gynecologists, and *Diagnosis and management of epilepsy in adults* from Healthcare Improvement Scotland [19–21]. Supplements include classification of epilepsy syndromes, surgical, psychological, immunological, dietary, and lifestyle treatments for epilepsy, treatment of comorbidities and complications of epilepsy, clinical neuropsychological and prognostic assessment of epilepsy, monitoring of therapeutic drug concentrations and regimen adjustment, menstrual cycle adjustment, monitoring of fetal health status, contraceptive measures, and perinatal substance supplementation. The data sources for the physical guideline annotations included 11 guideline documents on female epilepsy from 6 countries worldwide (UK, China, Canada, USA, Belgium, Spain) [22–29]. After identifying a total of 677 records through the initial database search, 154 duplicates were removed, leaving 523 articles for the initial screening based on titles and abstracts. Following this screening, 265 articles were deemed ineligible and excluded. Finally, after a thorough evaluation, 11 full-text articles were found to meet the inclusion criteria for our systematic review (see Fig. 2).

### Content of the ontology

The WWECA comprises a total of 609 entities organized within a hierarchical structure. The ontology’s hierarchy adheres to the architecture of the top-level ontology BFO (Basic Formal Ontology) to facilitate semantic interoperability between ontologies. It classifies entities into two primary categories: (1) Continuants: these encompass higher-level entities such as “disease,” “drug,” “diagnostic method,” “Etiology,” “Patient Health Management,” “Therapeutic Drug Monitoring,” “Contraception,” “Treatment plan adjustment,” and “Substance supplementation.” These categories serve as foundational building blocks for categorizing various aspects of the ontology. (2) Occurrences: The upper-level entity within this category is “Treatment process.” It represents dynamic, event-based aspects of the ontology, providing an overarching framework for understanding the processes and activities related to the management of women with epilepsy of child-bearing age.

**Fig. 3:**
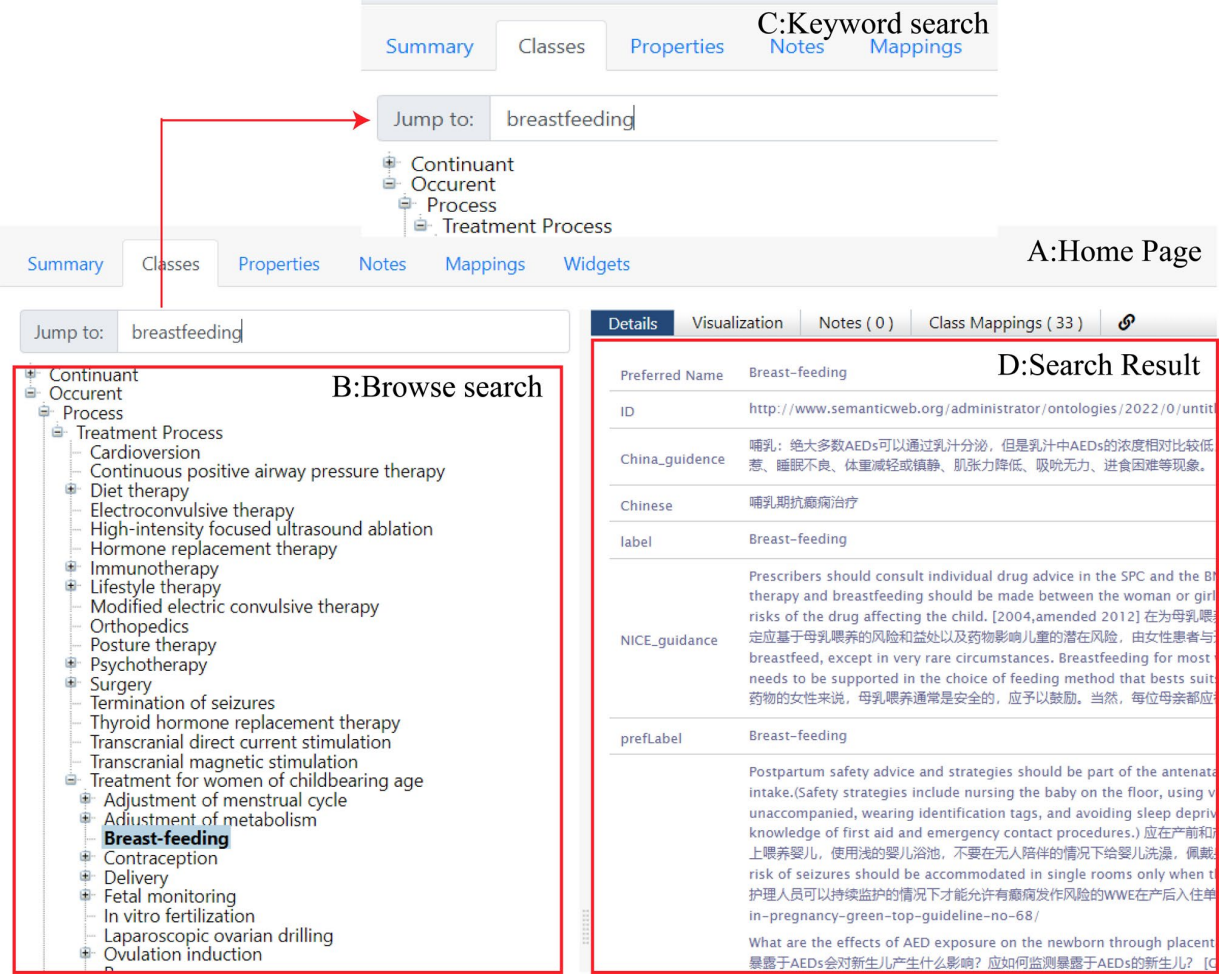
Search of ontology on Bioportal

As shown in Fig. 3, most of the terms are below the upper-level terms “disease,” “drug,” and “treatment process.” The maximum depth of the ontology is eight levels.Each entity is annotated with a preferred name (the most common preferred name for the item), a definition (a precise and short description of the basic characteristics or connotations of the entity, synonym, or abbreviation), synonyms or abbreviations (a group of words with the same meaning but different expressions) and a Chinese label.

The visual representation in Fig. 3 highlights that most of the terms within the ontology are descendants of the terms “disease,” “drug,” and “treatment process.“This distribution indicates a strong focus on these aspects within the ontology, reflecting their significance in managing women with epilepsy of childbearing age. Furthermore, the ontology exhibits a maximum depth of 8 levels, signifying its hierarchical structure’s capacity to encompass a diverse range of subcategories and entities, thus enabling a detailed and comprehensive information organization. Each entity within the ontology is associated with a set of annotations, which include:

- Preferred name: This denotes the entity’s most used name or label.
- Definition: A concise and precise description that outlines the fundamental characteristics or connotations of the entity, providing a clear understanding of its meaning and purpose.
- Synonyms or Abbreviations: These are alternative terms or expressions that convey the same or similar meanings as the preferred name. They enhance the accessibility and comprehensibility of the ontology by accommodating various ways of referring to a specific entity.
- Chinese Terminology: This attribute provides the Chinese-language equivalent for the entity, enabling users to access and understand the ontology in their preferred language.
- Chinese Terminology: This attribute provides the Chinese-language equivalent for the entity, enabling users to access and understand the ontology in their preferred language.

A total of 87.6% of the entities had synonyms from NCIm and MeSH, with the number of synonyms per entity ranging from 1 to 199. In addition, 91.0% of the entities had definitions from NCIm and MeSH. 18% of the entities also had the annotation of “guideline document”, referring to female epilepsy A sentence or paragraph in a guideline for patient management that addresses the entity.

### Application of ontology-driven online Q&A system

To facilitate ontology-driven knowledge translation, we use two web-based platforms to meet the needs of different users, such as healthcare professionals and researchers. The first is the public ontology repository Bioportal (Fig. 3A), which has a tree -structured navigation on the left-hand side of the ontology’s home page listing all entities and hierarchies of ontologies for women with epilepsy of childbearing age, allowing patients to search for entities both by browsing (Fig. 3B-C) and by keyword. As in the example in Fig. 3D, the user can quickly view the content of the guidelines related to ‘breastfeeding’.

The example of “breastfeeding” demonstrates the keyword and browsing search function for ontology on Bioprotal.

**Fig. 4:**
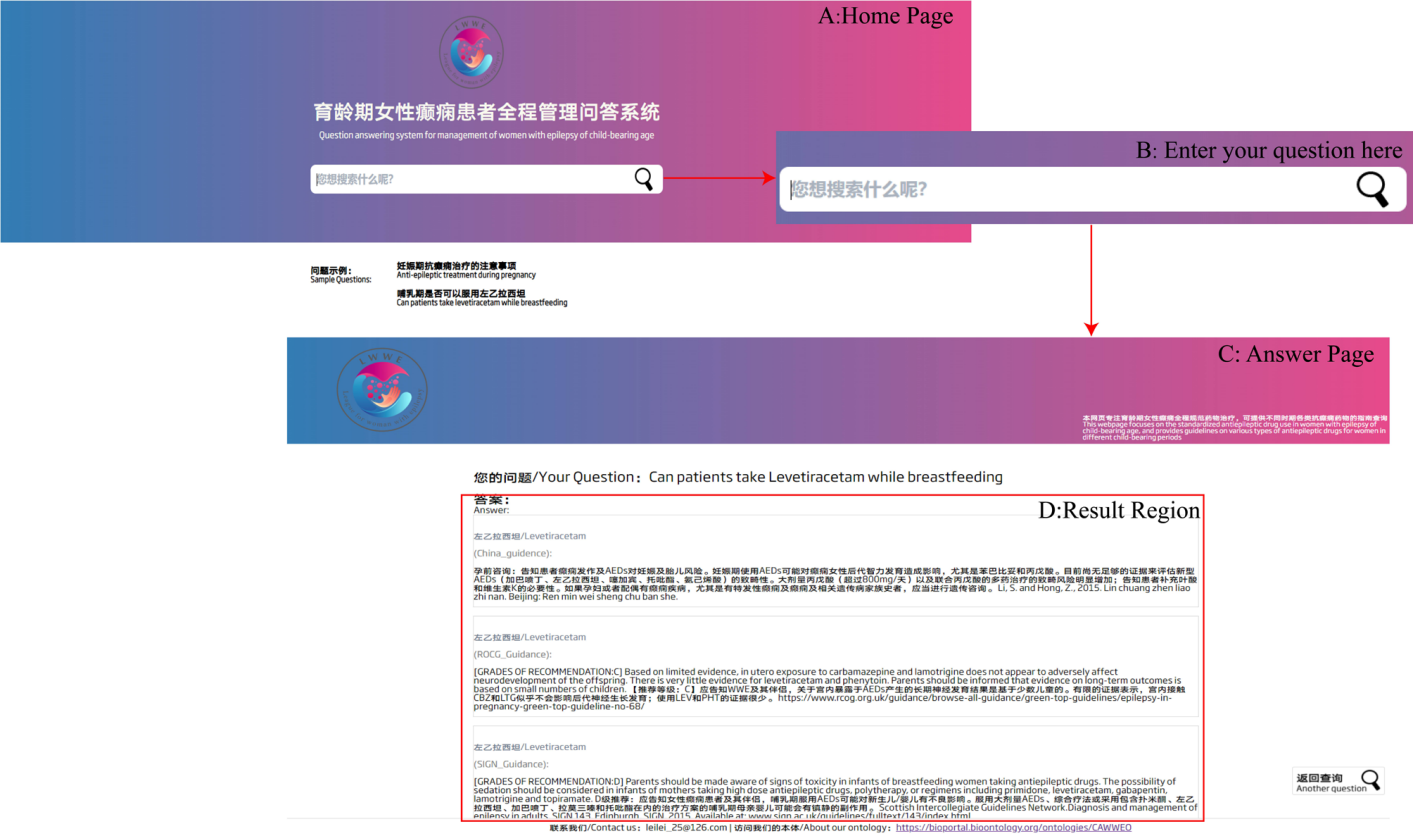
Ontology-driven online Q&A system

We also developed our own ontology-driven question and answer webpage for the full management of epilepsy patients of childbearing age, which supports bilingual queries (Fig. 4A). Users can ask questions directly in the input box (Fig. 4B). The webpage backend identifies named entities for the ASMs, period of reproductive age and adverse reaction entities and their synonyms in the question and provides feedback on the user’s answer based on the entity’s guideline annotations. An example scenario for the use of the question-and-answer system is shown in Fig. 4C.

The question “Is it possible to take levetiracetam while breastfeeding” is an example of how to ask and receive answers on the self-developed Q&A platform.

The framework for the web pages is TensorFlow. The operating system is Ubuntu 20.04 LTS. The private cloud infrastructure is Kubernetes and Docker, and the web server is Apache Tomcat v9.0.11.

**Fig. 5:**
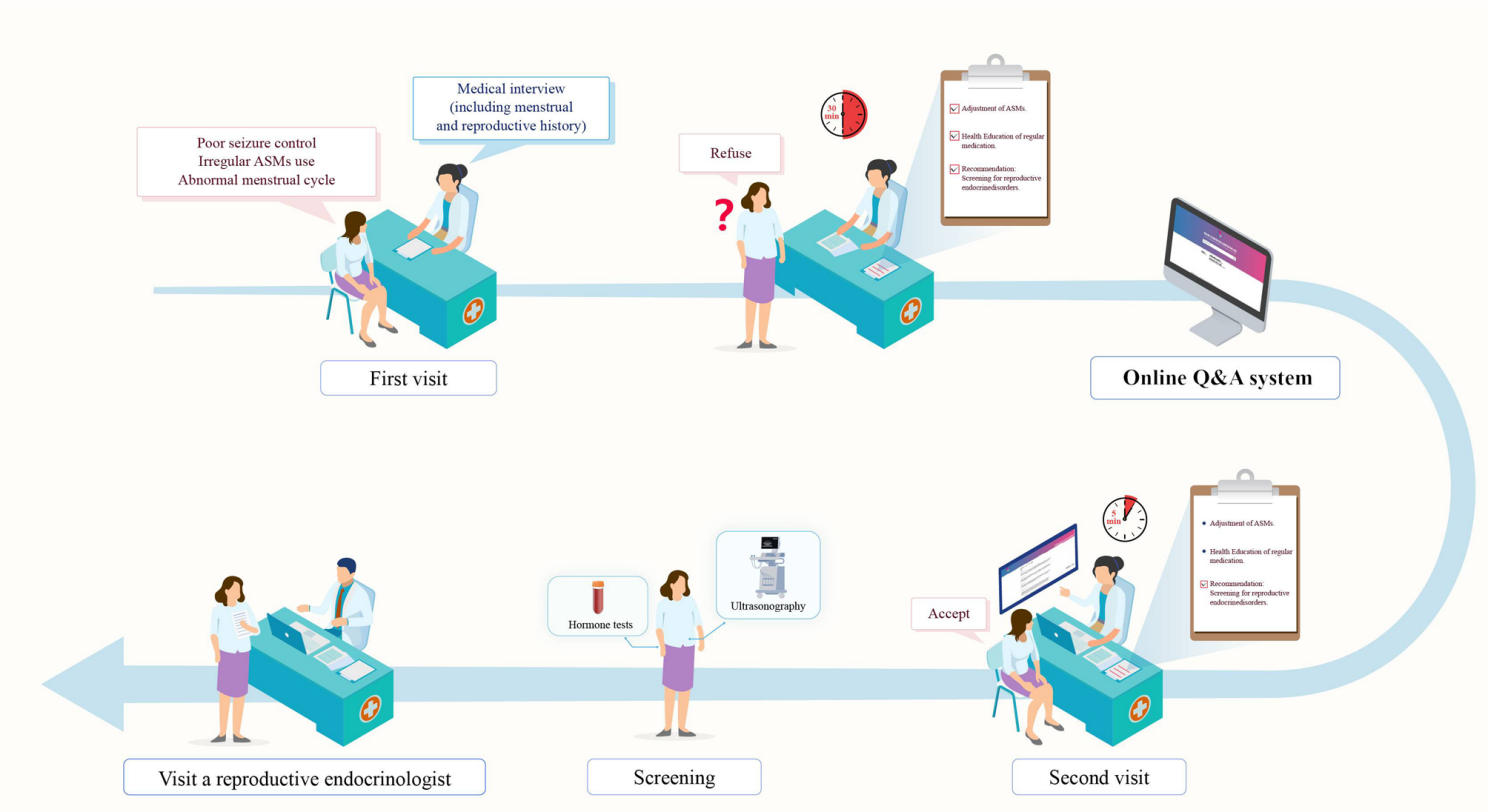
A case report of a patient using Q&A system. ASMs: Antiseizure medication; Q&A: Question and answer

During the utilization of the question-and-answer system, a noteworthy case emerged involving a 22-year-old woman with epilepsy, of child-bearing age, who presented with poorly controlled seizures (Fig. 5). In the context of a routine consultation regarding menstrual and fertility history, it was revealed that she experienced symptoms of menstrual irregularity and had no sexual history. After a thorough discussion, the suspicion arose that her seizures and ASMs may be contributing to these symptoms. Consequently, we recommended screening for reproductive endocrine disorders and a transition to a more regular medication regimen. Initially, the patient accepted the medication change but declined hormonal testing and ultrasound. Upon the implementation of the Q&A system, the patient’s approach evolved significantly. During a subsequent outpatient visit, she was presented with comprehensive information from authoritative sources, which led her to consent to hormonal testing. Subsequent assessment confirmed elevated testosterone levels, prompting a referral to the reproductive endocrinology unit for further evaluation. This case underscores the valuable role of the Q&A system in enhancing patient engagement, informed decision-making, and ultimately, healthcare outcomes.

### External assessment of WWECA

Throughout the development process, we regularly consulted experts to obtain dynamic adjustments.The guidelines and consensus documents we incorporated were also approved by experts.The simplicity, clarity and consistency of the ontology were confirmed by experts. In order to improve the accuracy of the ontology, the informaticians suggested that we ‘use the prefer names from the public authoritative databases as class’. Therefore, we renamed the names of 43 entities, including ‘Amniocentesis’ and ‘Contraceptive’.Obstetricians recommended that we add ‘Breastfeeding’ as a period to address the postnatal breastfeeding issue that is a major concern for mothers with epilepsy.

The first round of the rapid Delphi exercise included four epileptologists and one medical informatics expert; the second round included 35 epileptologists from 10 cities in China and achieved a better consensus on what was covered by the WWECA upper-level concept. The results of the second round are shown in Table 1. Consensus was obtained for all of the statements.

**Table 1:** Number of experts consulted in the second round of rapid Delphi exercise and percentage of agreement with the ontology superordinate entity

Statement	Strongly disagree, n	Disagree, n	Neither agree or disagree, n	Agree, n	Strongly agree, n	% Agreement
Disease	0	0	2	20	13	94.3
Etiology	0	0	1	7	27	97.1
Patient care management	0	0	4	10	21	88.6
Therapeutic drug monitoring	0	0	3	6	26	91.4
Adjustment of treatment plan	0	0	2	8	25	94.3
Contraceptive	0	0	6	8	21	82.9
Diagnosis method	0	0	2	4	29	94.3
Material supplement	0	0	4	10	21	91.1
Medication	0	0	0	10	35	88.6
Treatment Process	0	0	2	9	24	94.3

We compared the WWECA vocabulary with and SNOMED CT, and the results showed that 83% (*n* = 505) of the vocabulary came from public sources with high domain credibility.Inconsistency and unfulfillability are checked using the HermiT reasoner (0.145 s). If any errors occur, they are usually corrected until the reasoner does not provide any discrepancies. Thus, the accuracy of the vocabulary, the rationality of the structure, the diversity of the semantic relations, and the feasibility of the application meet the requirements for ontology construction(sTable 2).

Participants in the trial and evaluation of the website included five neurologists and five obstetricians and gynecologists from five centers in Southwest China, including interns (*n* = 1), residents (*n* = 4) and attending physicians and above (*n* = 5) involved in the management of epilepsy in women of childbearing age, as well as Chinese (*n* = 8) and English (*n* = 2) users. They evaluated the Q&A system in five areas: overall experience of use, knowledgeability, usability, ability to assist in clinical decision-making, and promotion of a harmonious doctor‒patient relationship. The results of the scores are shown in Table 2. The overall experience of using the Q&A webpage received a mean rating of 4.2 (SD 0.6) among physicians.

**Table 2:** Results of satisfaction survey among neurologists and obstetricians and gynecologists with the question and answer system for the full management of women with epilepsy in the reproductive age group

Questions	Neurologists (SD)	Obstetricians and Gynecologists (SD)	Total (SD)
What extent do you satisfied with your experience of using the Q&A website	4.4(0.6)	4.4(0.5)	4.2(0.6)
What extent do you satisfied with the knowledgeability Q&A website	4.4(0.8)	4.6(0.5)	4.5(0.7)
What extent do you satisfied with the usability Q&A website	3.8(0.7)	4.2(0.7)	4.0(0.8)
Does the Q&A website help you with clinical decision-making	4.2(0.7)	4.2(0.7)	4.2(0.7)
Does the Q&A website help you to promote a harmonious doctor-patient relationship	3.8(0.7)	4.2(0.4)	4.0(0.6)

## Discussion

**Fig. 6:**
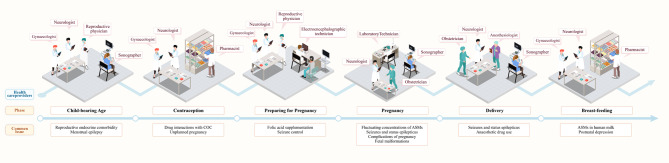
Multidisciplinary teams of health care providers and common issues of whole-cycle management WWE. COC: Combined oral contraceptive; ASMs: Antiseizure medication

Existing ontologies in the domain of epilepsy have played a significant role in patient management (Table 3). However, prior epilepsy ontologies fell short in delivering a comprehensive and in-depth representation of the specific area of WWE management during their child-bearing years. The intricate management of women with epilepsy (WWE) during their child-bearing years demands an integrated approach involving multiple healthcare disciplines, as exemplified in Fig. 6. Even in the early stages of multidisciplinary teams (MDTs), obstacles associated with disciplinary boundaries can result in a siloed approach, necessitating prolonged efforts in mutual education and interdisciplinary cooperation.

**Table 3:** Existing ontologies of epilepsy

Ontology	BioPortal Acronym	Year	Number of entities	Content	Application	Author	language	Downloads in the last year
Epilepsy Ontology	EPILONT	2011	138	EEG, classification, symptoms, complications, treatment, epilepsy syndrome, etc.	European Epilepsy Database and Epilepsy Alert Device [30]	Antonio Dourado et al.	Portuguese English	20
Epilepsy and Seizure Ontology [18]	EPSO	2013	1,933	Etiology, classification, patient management, clinical and neurophysiology, molecular pathology, ASMs, anatomical description of epileptogenic foci, genes and their variants	Epilepsy self-management platform [31] Natural language processing based text data extraction [32] Multicenter data integration system for sudden death in epilepsy [33] Electrophysiological data visualization and analysis platform [34] Information capture system for epilepsy medical record data [35] Multicenter data integration system for sudden death in epilepsy [36] Extraction of epileptic phenotypes and their associated anatomical locations from discharge medical records [37]	Satya S Sahoo et al.	English	264
Epilepsy Syndrome Seizure Ontology	ESSO	2015	2,705	Clinical symptoms, classification, diagnosis and differential diagnosis, epidemiology, etiology, prognosis and treatment	ESSO-based ontology of epilepsy symptomatology	Robert Yao et al.	English	162
Epilepsy Semiology	EPISEM	2019	1,594	Symptomatology, including seizure and post-ictal, interictal and aura symptomatology	-	Daniel Hier et al.	English	60
Functional Epilepsy Nomenclature for Ion Channels	FENICS	2020	153	Function and physiological characteristics of epilepsy-related ion channels and their interactions	-	Ingo Helbig et al.	English	162

Our study addressed this gap by amalgamating guidelines and expert opinions to create the world’s inaugural ontology in the realm of WWE. This ontology has undergone evaluation and by epileptologists from ten medical centers and semi-structured interviews with multidisciplinary team doctors, ensuring a more thorough coverage of pivotal aspects concerning women of child-bearing age throughout their various life stages. To amplify the utility of this ontology in clinical settings, we have concurrently developed an ontology-based question-and-answer system. Remarkably, this system has garnered a high level of satisfaction in multidisciplinary assessments, thereby affirming its effectiveness in addressing the unique needs and inquiries of healthcare providers and researchers within this specialized domain.

Our surveys in the fields of neurology and obstetrics and gynecology have underscored the potential of question-and-answer webpages in improving patient compliance and strengthening doctor-patient relationships. An illustrative case revealed a notable cognitive and attitudinal transformation in a reproductive-age woman with epilepsy before and after her interaction with the webpage. It is worth noting that this patient had polycystic ovary syndrome, the most prevalent reproductive endocrine comorbidity in epilepsy [38]. Initially, she displayed hesitation regarding relevant examinations, such as serum hormone testing and ultrasound, primarily due to their non-routine nature in her region. However, her trust increased during a subsequent visit, attributable to the webpage’s knowledge source’s credibility and the multimedia approach adopted by the specialist. This transformation signified a swifter acceptance of advanced management concepts surpassing conventional textbook knowledge. Nonetheless, it’s important to consider a certain limitation when interpreting the case. Patient engagement was missing from the development of the WWECA ontology. Patient and public involvement is already a key part of clinical practice guideline development, but has not yet been fully applied in ontology development [15]. In addition, patients with different languages, cultures, healthcare systems, and educational backgrounds may respond very differently to the same decision support tool, which needs to be further evaluated in the future [39].

There are several other limitations to our study. First, WWECA and the Q&A system have not been integrated with electronic health records for decision support such as medication recommendations for WWE at different times. Second, our online Q&A system is single-device use with guideline text recommendations and lacks the application of multi-media, multi-device, and more interactive technologies.

## Conclusion

The development of WWECA and the Q&A webpage holds promise as a versatile tool for managing complex, long-term health conditions. This model may extend to the management of other chronic diseases. Our development team envisions that as WWECA is promoted and adopted globally, experts and scholars can adapt and enhance its domain knowledge to better serve women with epilepsy of child-bearing age based on their cultural and technological contexts.

### Electronic supplementary material

Below is the link to the electronic supplementary material.

Supplementary Material 1

## Data Availability

Data resource access: https://bioportal.bioontology.org/ontologies/WWECA. http://101.207.6.21:9030/search/.
